# In Vitro Influence of Extracts from Snail *Helix aspersa* Müller on the Colon Cancer Cell Line Caco-2

**DOI:** 10.3390/ijms19041064

**Published:** 2018-04-03

**Authors:** Magdalena Matusiewicz, Iwona Kosieradzka, Tomasz Niemiec, Marta Grodzik, Hanna Antushevich, Barbara Strojny, Małgorzata Gołębiewska

**Affiliations:** 1 Department of Animal Nutrition and Biotechnology, Faculty of Animal Sciences, Warsaw University of Life Sciences, Ciszewskiego 8, 02-786 Warsaw, Poland; iwona_kosieradzka@sggw.pl (I.K.); tomasz_niemiec@sggw.pl (T.N.); marta_grodzik@sggw.pl (M.G.); barbara_strojny@sggw.pl (B.S.); golebiewskamalgorzata2@gmail.com (M.G.); 2 Laboratory of Molecular Biology, the Kielanowski Institute of Animal Physiology and Nutrition, Polish Academy of Sciences, Instytucka 3, 05-110 Jabłonna, Poland; antuszewicz@op.pl

**Keywords:** *Helix aspersa* Müller, tissue extracts, chemical composition, colon cancer, Caco-2

## Abstract

Colorectal cancer is the third most widely diagnosed cancer. Extracts from snails may modulate growth and development of colorectal cancer cells. The objective of this study was to determine the chemical composition of tissues derived from *Helix aspersa* Müller and red-ox properties of tissue extracts. Then, the influence of extracts and their fractions of different molecular weights on viability of Caco-2 cells was examined. Tissue lyophilisates contained antioxidants that could be important in the prevention of colorectal cancer. Moreover, we confirmed the presence of a wide array of compounds that might be used in treatment of this disease. The decrease of cell viability after the application of extracts from lyophilized mucus and foot tissues was affirmed. The effect of extract from mucus could be related to the content of some proteins and peptides, proper essential amino acids (EAA)/non-essential amino acids (NEAA) ratio, Met restriction and the presence of Cu, Ca, Zn, Se. The influence of the extract from foot tissues could be assigned additionally to the presence of eicosapentaenoic, α-linolenic, linoleic and γ-linolenic acids. The opposite effect was demonstrated by extract from lyophilized shells which increased cell viability. Further studies are needed to know whether dietary supplying of *H. aspersa* Müller tissues can be used as an approach in colorectal cancer management.

## 1. Introduction

Colorectal cancer is the third most widely diagnosed cancer and the fourth cause of cancer death worldwide [1]. The distribution of colorectal cancer is varied; more than two-thirds of cases and about 60% of deaths occurred in countries having high or very high human development index. The global burden of colorectal cancer is anticipated to increase by 60% by 2030. Its complex etiology involves genetic and environmental factors, with the most important probably being diet [2]. Conventional treatment of colorectal cancer mainly consists of surgical resection with or without radiation therapy and/or chemotherapy [3]. Even though, this treatment is often ineffective. Over half of sick people develop local relapses or metastasis in two years after surgery. Different modalities to improve treatment of colorectal cancer are of great interest. An innovative way to decrease its occurrence may be dietary intervention.

There are increasing evidences that the important sources of natural drugs exhibiting anticancer activities could be different edible plant species and invertebrates, land and water, including mollusks. Mollusks are about a hundred thousand species and form the second largest phylum in animal kingdom [4]. One category of the univalve organisms among mollusks is that of snails. They have been consumed for thousands of years in many countries, however, until now, information about the nutritional value and chemical composition of edible parts of snails have been limited. One of the most consumed land snail species is the common garden snail *Helix aspersa* Müller.

Extracts from tissues of *H. aspersa* Müller were shown to possess anticancer activity against breast cancer cells (Hs578T) [5]. Two fractions of mucus of another snail, the giant African snail *Achatina fulica*, decreased viability of breast cancer cells (MCF-7) [6]. In turn, the mucus of *Actinia equina* showed cytotoxic effect on human erythromyeloid leukemia-derived cells (K592) [7]. Hemocyanins, oxygen-carrying hemolymph metalloproteins, and their isoforms from the land snail *Helix lucorum* and marine snail *Rapana venosa* exhibited anticancer effects on human bladder carcinoma cells, T-24 [8]. Hemocyanins derived from *H. aspersa* Müller and *H. lucorum* and structural subunit from *H. aspersa* Müller hemocyanin decreased viability of bladder cancer cells (T-24 and CAL-29) [9]. Moreover, hemocyanins isolated from *H. aspersa* Müller decreased the viability of ovarian cancer cells (FraWü), prostate cancer cells (DU-145), acute monocytic leukemia cells (THP-1), Burkitt’s lymphoma cells (Daudi) and glioma cancer cells (LN-18). Hemocyanins which were isolated from the land snail *Helix pomatia* and marine snail *Rapana thomasiana* expressed strong anticancer and antiproliferative action in a murine model of colon carcinoma [10]. The immunization with hemocyanins prolonged survival, increased the humoral anticancer response and moderated tumor growth and lung metastasis. Lectins from snails have been applied as markers for metastatic tissues in colon and breast cancers [11,12]. 

Considering the above, the extracts from snail tissues containing chemical compounds with potential anticancer properties may modulate growth and development of colorectal cancer cells. The objective of this study was to determine the chemical composition of tissues derived from *H. aspersa* Müller—mucus, foot tissues and shells, as well as to evaluate red-ox properties of extracts obtained from these tissues. Then, the influence of compounds contained in snail extracts and the effect of extract fractions of different molecular weights on the viability of human colorectal adenocarcinoma Caco-2 cells were examined.

## 2. Results

### 2.1. Red-Ox State Indicators

**Figure 1:**
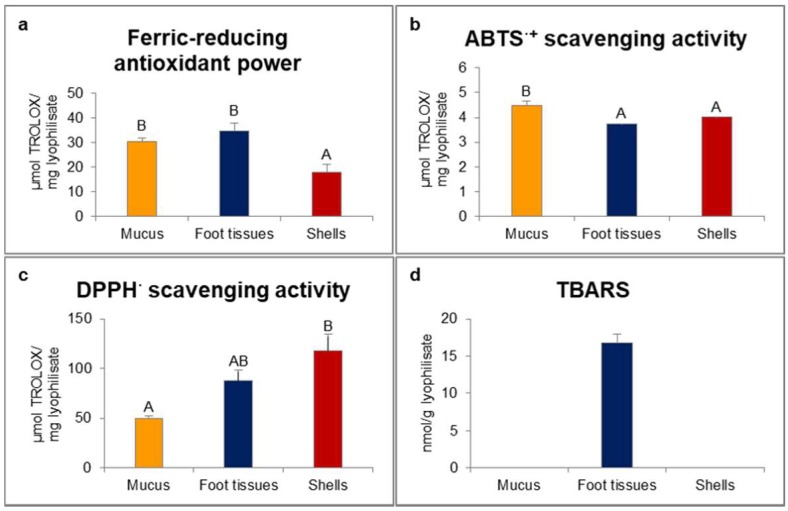
Red-ox state indicators: (**a**) ferric-reducing antioxidant power; (**b**) ABTS*^·^*^+^ (2.2′-azino-bis(3-ethylbenzthiazoline-6-sulfonic acid) radical cation) scavenging activity; (**c**) DPPH^·^ (2.2-diphenyl-1-picrylhydrazyl radical) scavenging activity; (**d**) TBARS (thiobarbituric acid reactive substances) of extracts from mucus, foot tissues and shells of *Helix aspersa* Müller. Error bars indicate standard error of the mean (SEM). Statistically significant effect: values of one marker without common superscript (**A**,**B**) are statistically significantly different (*p* < 0.01). *n* = 6.

In our study, ferric-reducing antioxidant power was statistically significantly higher for mucus and foot tissues of *H. aspersa* Müller compared to shells (Figure 1). Scavenging activity of 2.2′-azino-bis(3-ethylbenzthiazoline-6-sulfonic acid) radical cation (ABTS*^·^*^+^) was significantly the highest for mucus. In turn, scavenging activity of 2.2-diphenyl-1-picrylhydrazyl radical (DPPH^·^) was significantly the lowest for mucus and the highest for shells. Only in foot tissues were detected thiobarbituric acid reactive substances (TBARS).

### 2.2. Proximate Composition

The data showed that foot tissues of snails consisted mostly of crude protein, the level of which was higher than in mucus and much higher than in shells (Table 1). Furthermore, the mucus and shells scarcely contained crude fat, whereas foot tissues contained little amounts, less than 4%. The crude ash content was the highest in shells of snails and the lowest in foot tissues.

### 2.3. Analysis of Molecular Weights of Proteins

**Figure 2:**
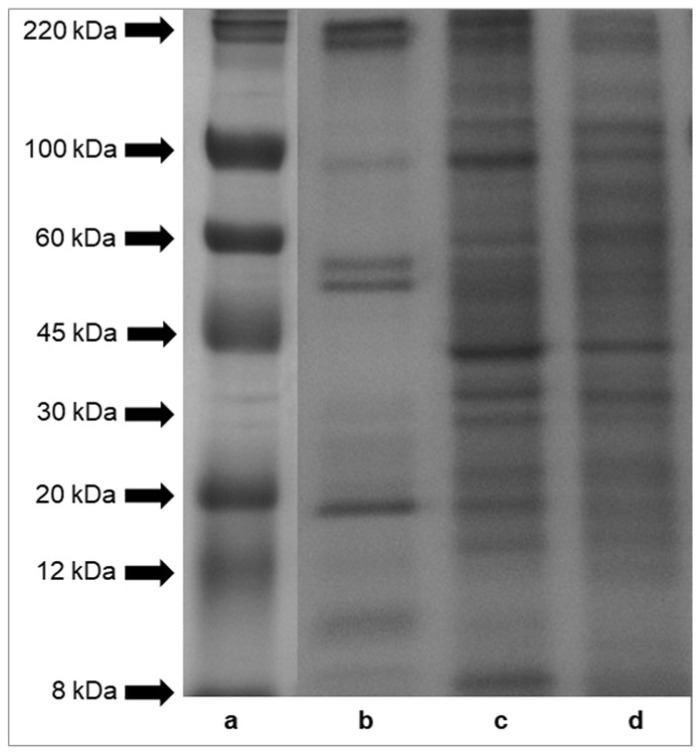
SDS-PAGE analysis of proteins isolated from *Helix aspersa* Müller body parts. Panel (**a**)—molecular weights of standard proteins furnished by Sigma-Aldrich, Inc.; panel (**b**)—mucus extract; panel (**c**)—foot tissues extract and panel (**d**)—shell extract.

Sodium dodecyl sulfate-polyacrylamide gel electrophoresis (SDS-PAGE) profiles of proteins of mucus, foot tissues and shells of *H. aspersa* Müller are shown in Figure 2. The results indicated that protein bands of tissues had molecular weights across the range of standard proteins, from 8 to 220 kDa. A comparison of the protein patterns revealed that the protein components of foot tissues and shells (panel (c) and (d)) were more numerous than those of mucus (panel (b)). The electrophoresis analysis showed six major protein bands of mucus, with a molecular weight of about 220 kDa, about 100 kDa, two bands >50 kDa, <20 kDa, about 10 kDa. Bands of proteins and peptides below the 8 kDa range were also observed in case of all analyzed tissues.

### 2.4. Analysis of Amino Acids

The profiles of amino acids in different tissues of *H. aspersa* Müller are shown in Table 2. Eighteen different amino acids of which eight were essential amino acids (EAA) for humans, including isoleucine (Ile), leucine (Leu), lysine (Lys), methionine (Met), phenylalanine (Phe), threonine (Thr), valine (Val) and tryptophan (Trp) were identified [13]. The amount of individual amino acids in crude protein differed among various tissues of snails. Amino acid score (AAS), chemical score (CS) and essential amino acid index (EAAI) for mucus, foot tissues and shells of *H. aspersa* Müller are presented in Table 3. AAS for mucus, for His (histidine), Lys, Trp and AAS for foot tissues, for His, Leu, Lys, Trp, Met + Cys (cysteine) were <1.00. According to the AAS, the first limiting amino acids were Lys for mucus, Trp for foot tissues and His for shells. The levels of Ile, Thr and Val in the three examined snail tissues were the highest in comparison to FAO (the Food and Agriculture Organization of the United Nations, Roma, Italy)/WHO (the World Health Organization, Geneva, Switzerland) reference amino acid pattern. CS for amino acids of mucus and foot tissues were <1.00. According to the CS, the first limiting amino acids were Trp and Met + Cys for mucus and Met + Cys for foot tissues and shells. The content of Met in mucus was very low compared to the other amino acids and compared to its amount in other tissues (Table 2). The EAAI (FAO/WHO reference protein) was slightly lower than 100 only for foot tissues. The EAAI (whole egg reference protein) was <100 for mucus and foot tissues.

Among the non-essential amino acids (NEAA), glutamic acid (Glu), aspartic acid (Asp) and glycine (Gly) were found to be the most abundant in all tissues.

Our study demonstrated that all tissues of *H. aspersa* Müller contained high levels of delicious amino acids (DAA)—Glu, Asp, Gly and Ala (alanine).

DAA/TAA (total amino acids) ratio was the highest for foot tissues and shells. Generally, it was noted that the ratios EAA/TAA and EAA/NEAA were the highest for mucus and shells (EAA/TAA—0.39 and 0.40, respectively; EAA/NEAA—0.74 and 0.75, respectively). EAA/TAA for foot tissues was 0.34 and EAA/NEAA—0.62. 

### 2.5. Composition of Fatty Acids

The fatty acid profile of *H. aspersa* Müller mucus was not determined because of the very low fat content. The compositions of fatty acids of foot tissues and shells are shown in Table 4. In total, 23 fatty acids were quantified (contents of individual fatty acid methyl esters (FAME) ≥0.050 g/100 g FAME) in foot tissues and 25 in shells, in the present study. 7 saturated fatty acids (SFA) were determined quantitatively in foot tissues, 5 monounsaturated fatty acids (MUFA) and 11 polyunsaturated fatty acids (PUFA), including *trans* isomers. In turn, in shells, we quantified 10 SFA, 5 MUFA and 10 PUFAs. Significant differences (*p* < 0.05 or *p* < 0.01) in the concentrations of individual fatty acids between the two examined snail tissues were noted except for palmitic acid, γ-linolenic acid, behenic acid and tricosylic acid. The composition of fatty acids of foot tissues was 25.9 ± 0.4 g SFA/100 g FAME, 27.0 ± 0.3 g MUFA/100 g FAME and 33.6 ± 0.5 g PUFA/100 g FAME, whereas that of shells differed statistically significantly and was 11.9 ± 0.8 g SFA/100 g FAME, 35.3 ± 1.4 g MUFA/100 g FAME and 35.7 ± 0.6 g PUFA/100 g FAME.

Among SFA, stearic and palimitic acids were the most abundant in foot tissues and shells (foot tissues—16.27 ± 0.03 g/100 g FAME and 5.02 ± 0.33 g/100 g FAME, respectively; shells—4.71 ± 0.22 g/100 g FAME and 5.20 ± 0.40 g/100 g FAME, respectively). In shells was quantified (0.227 ± 0.004 g/100 g FAME) myristic acid.

Our results revealed statistically significant differences among the sum of three quantified n-3 PUFA concentrations in foot tissues and shells which were 6.65 ± 0.20 g/100 g FAME and 3.85 ± 0.07 g/100 g FAME, respectively. The content of α-linolenic acid was 2.74 ± 0.21 g/100 g FAME in foot tissues and 3.63 ± 0.09 g/100 g FAME in shells; of eicosatrienoic acid was 0.584 ± 0.114 g/100 g FAME in foot tissues and 0.053 ± 0.006 g/100 g FAME in shells and of eicosapentaenoic acid (EPA) was 3.323 ± 0.141 g/100 g FAME in foot tissues and 0.169 ± 0.009 in shells.

The content of n-6 PUFA sum differed significantly between foot tissues and shells and were 26.5 ± 0.3 g/100 g FAME and 31.4 ± 0.5 g/100 g FAME, respectively. The concentration of linoleic acid was 16.4 ± 0.5 g/100 g FAME in foot tissues and 28.1 ± 0.6 g/100 g FAME in shells; of γ-linolenic acid was 0.091 ± 0.018 g/100 g FAME in foot tissues and 0.101 ± 0.007 g/100 g FAME in shells; of eicosadienoic acid was 8.92 ± 0.46 g/100 g FAME in foot tissues and 2.62 ± 0.07 g/100 g FAME in shells; of dihomo-γ-linolenic was 0.977 ± 0.198 g/100 g FAME in foot tisses and 0.609 ± 0.006 g/100 g FAME in shells. Docosadienoic acid was quantified only in foot tissues (0.068 ± 0.013 g/100 g FAME).

Moreover, foot tissues and shells were significantly different in n-6/n-3 ratio (3.98 ± 0.09 and 8.15 ± 0.03, respectively).

Both tissues turned out to contain little *trans* fatty acids (TFA).

### 2.6. Analysis of Minerals

The macroelements and microelements detected in mucus, foot tissues and shells of *H. aspersa* Müller are presented in Table 5. The contents of Cl, S and Si were not determined in mucus because of the lack of enough amount of material. The concentrations of minerals, except Se, differed statistically significantly (*p* < 0.01) between snail tissues. The decreasing contents of macroelements in mucus were as follows: Na, Ca, K, Mg, P; in foot tissues: Ca, K, P, Na, S, Cl, Mg and in shells: Ca, P, K, S, Na, Mg, Cl. The decreasing contents of microelements in mucus were as follows: Cu, Zn, B, Fe, Mo, Mn, Cr, Ni, Se, Co; in foot tissues: Si, Fe, Zn, Cu, Mn, Ni, B, Mo, Cr, Se, Co and in shells: Fe, Si, Mn, Zn, Cu, Ni, B, Co, Cr, Mo.

### 2.7. Influence of Extracts on Cell Viability

**Figure 3:**
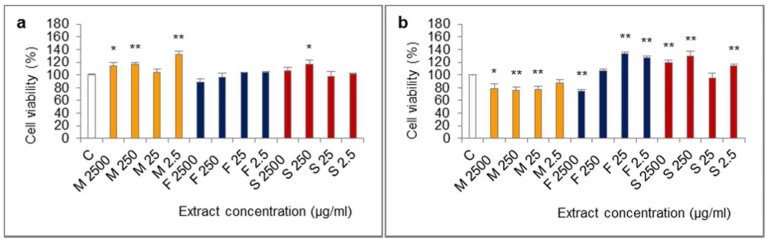
Viability of Caco-2 cell line after (**a**) 24 h and (**b**) 72 h of treatment with extracts from mucus (M), foot tissues (F) and shells (S) of *Helix aspersa* Müller, at the concentrations of 2500, 250, 25, 2.5 µg/mL (2500, 250, 25, 2.5, respectively). C—control cells. Error bars indicate standard error of the mean (SEM). Statistically significant effect: * represents values that differ from control at *p* <0.05, ** represents values that differ from control at *p* <0.01. *n* = 6.

The influence of extracts from mucus, foot tissues and shells of *H. aspersa* Müller, at different concentrations, on the viability of Caco-2 cells was evaluated in the MTT (methylthiazolyldiphenyl-tetrazolium bromide) assay, in which the number of living, metabolically active cells is proportional to the amount of reduced colorful MTT and oxidative activity of cell mitochondria. The results of the 24-h test showed that all of the applied levels of extract from mucus increased viability of cells, the effect was statistically significant for 2500, 250 and 2.5 μg/mL (Figure 3). After 24 h of cell treatment with the extract from foot tissues at 2500 μg/mL, viability was reduced, however, the difference was not statistically significant (*p* = 0.0592). In turn, extract from shells at 250 μg/mL significantly inceased viability.

The opposite situation was noted in case of viability of Caco-2 cells which were treated with every concentration of the extract from mucus for 72 h. Viability of these cells was lower than control and was 76–78% of the viability of controls, for 2500, 250 and 25 μg/mL (statistically significant effect) and for 2.5 μg/mL the effect was at the border of significance (*p* = 0.0576). In the 72-h test, like in the 24-h test, viability of cells after treatment with the extract from foot tissues at 2500 µg/mL was reduced and the difference was statistically significant. In turn, the concentrations of 25 and 2.5 µg/mL of this extract caused a significant increase of viability. After 72 h of treatment with the extract from shells at 2500, 250 and 2.5 µg/mL, viability was significantly higher. 

### 2.8. Influence of Fractions of Extracts on Cell Viability

**Figure 4:**
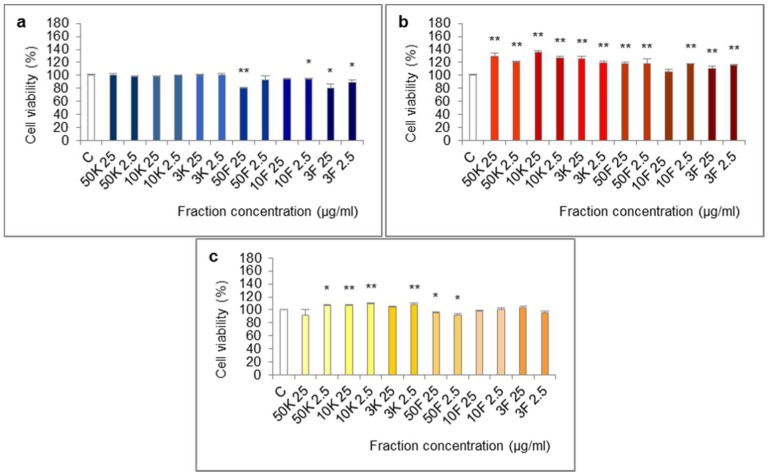
Viability of Caco-2 cell line after treatment with fractions >50 kDa (50K), 10–50 kDa (10K), 3–10 kDa (3K), <50 kDa (50F), <10 kDa (10F) and <3 kDa (3F) of extracts from different body parts of *Helix aspersa* Müller, at the concentrations of 25 and 2.5 µg/mL (25 and 2.5, respectively). (**a**,**b**)—cell viability after treatment for 24 h with fractions of extracts from foot tissues and shells, respectively; (**c**) cell viability after treatment for 72 h with fractions of extract from mucus. C—control cells (treated with deionized water). Error bars indicate standard error of the mean (SEM). Statistically significant effect: * represents values that differ from control at *p* < 0.05, ** represents values that differ from control at *p* < 0.01. *n* = 6.

The molecules of molecular weight <50 kDa, <10 kDa and <3 kDa of extract from foot tissues reduced the viability of Caco-2 cells in the 24-h test (Figure 4). The effect was statistically significant for fractions <50 kDa (25 µg/mL), <10 kDa (2.5 µg/mL), <3 kDa (25 and 2.5 µg/mL) and at the border of statistical significance for fraction <10 kDa (25 µg/mL, *p* = 0.0569).

All of the fractions of extract from shells increased cell viability in the 24-h test. The differences were statistically significant except for fraction <10 kDa at 25 µg/mL.

In the 72-h test, some of the fractions of extract from mucus: >50 kDa (2.5 µg/mL), 10–50 kDa (25 and 2.5 µg/mL), 3–10 kDa (2.5 µg/mL) significantly increased cell viability and fraction <50 kDa (25 and 2.5 µg/mL) significantly reduced it.

## 3. Discussion

Long-term antioxidants ingestion could reduce the incidence of colorectal cancer in rats, by decreasing oxidative stress [14]. Antioxidants could also improve severity of colitis, inflammation and the preneoplastic state of colorectal cancer, by decreasing elevated malondialdehyde (MDA) levels. In our investigation, ferric-reducing antioxidant power, representing the electron donor properties of antioxidants for neutralizing free radicals by formation of stable products [15], was the highest for mucus and foot tissues of *H. aspersa* Müller. ABTS^·^^+^ scavenging activity, also based on an electron-transfer capacity, was the highest for mucus. In turn, scavenging activity of DPPH^·^, being a stable free radical which accepts an electron or hydrogen radical becoming a stable molecule, was the lowest for mucus and the highest for shells. The reason for the observed differences could be the fact that hydrophilic and high-pigmented antioxidants are better reflected by ABTS than DPPH assay [16]. Moreover, some compounds which were analysed in ABTS assay could have required considerably longer time of incubation to reach the end-point [17].

Peroxidation of PUFA contributes to oxidative stress augmentation and in consequence, inflammation, one of the factors of colorectal cancer development [14]. Lipid peroxidation provokes disruption of cell membranes and generation of reactive carbonyl compounds, alkanes and ketones. These compounds produce end products among which the most mutagenic is MDA and the most toxic is 4-hydroxy-2-nonenal (4-HNE). The end products might be used for detection of colorectal cancer and surveillance for treatment. MDA reacts with nucleic acid bases and forms adducts which can cause apoptosis or induce genetic mutations. Moreover, MDAs with 4-HNE form adducts with elongation factor two in ribosome that consequently reduce or disturb protein synthesis, often observed during the development of cancer. Toxic properties of 4-HNE are related to its high chemical reactivity and long half-life clearance. It mainly reacts with proteins and tyrosine kinase-associated receptors, but also causes severe DNA damage. All of these lead to modification of cell signaling, cell cycle and inhibition or activation of related enzymes, that would be carcinogenic. 4-HNE inhibits cell growth, its low concentration triggers cell proliferation. 4-HNE is a potential mitochondrial permeability transition mediator; it reacts with calcium ATP-ase, that can disrupt homeostasis of calcium and induce cell death. 4-HNE could increase oxidative stress, promote cellular glutathione consumption, mediated by glutathione S-transferase and by Se-dependent glutathione peroxidase inactivation. 4-HNE, mediated by the above mechanisms, modifies the activity of tumor suppressor protein which is associated with a chronic infammatory state.

4-HNE could inhibit growth of colorectal cancer Caco-2 and HT-29 cell lines. The anticancer effect might be connected with alterations of oxidative stress and consequent induction of apoptosis and inhibition of telomerase activity. Glutathione conjugates of 4-HNE are already used in some cancer models as chemotherapeutic agents. In our study, products of lipid peroxidation were detected only in foot tissues.

The crude protein content in lyophilised foot tissues of snails was the highest among the examined tissues and was similar to the content in dry matter of beef [18,19]. About 65 g of snail foot tissues lyophilisate covers daily protein requirement of adult of 80 kg body weight [20]. In the study of Hu et al., due to the fact that cancer cells heavily rely on glycolysis, high protein and low carbohydrate diets were designed to see if blood glucose and tumor growth could be limited [21]. Both human colorectal carcinoma and murine squamous cell carcinoma VII grew slower in mice receiving high protein and low carbohydrate diets in comparison with a Western diet, low in protein and high in carbohydrates. Farther, snail tissues contained little amount of crude fat. The acceptable macronutrient distribution range (AMDR) for intake of total fat ranges from 20% to 35% of energy (E) [22].

The protein bands of snail tissues had molecular weights from 8 to 220 kDa. Bands below the 8 kDa range of the ladder were also present. In mucus, we identified six major bands, with a molecular weights of about 220 kDa, about 100 kDa, two bands >50 kDa, <20 kDa, about 10 kDa.

The study of Teerasak et al. revealed that two HPLC-separated fractions of *Achatina fulica* mucus decreased viability of mammary gland adenocarcinoma cells (MCF7) and epithelial cells (Vero) [6]. In fractions, were identified 16 putative cationic and amphipathic anticancer peptides. The majority of these peptides (9) were smaller than 3 kDa, 3 peptides had molecular weights 3–10 kDa, 2 of them were 10–50 kDa and 2 were bigger than 50 kDa. The anticancer peptides consisted of 5–25 amino acids. In the first HPLC fraction, 4 peptides were short-length and 2 random-coiled; in the second fraction were identified 1 single helix, 1 helix-consisted loop, 1 β-sheet-consisted loop, 4 random-coiled and 3 short peptides. It was proved that α-helical cationic anticancer peptides exhibit unique mechanisms of disruption of cytoplasmic membrane, which lead to necrosis and induction of apoptosis by interruption of the mitochondrial membrane. 

Availability of EAA is the main limiting factor of synthesis of proteins, because EAA enable endogenous formation of NEAA [23]. AAS for His, Lys and Trp in mucus and AAS for His, Leu, Lys, Trp and Met + Cys in foot tissues were <1.00, which demonstrates that these amino acids concentrations were lower than amino acids in FAO/WHO reference pattern [24]. According to the AAS, the first limiting amino acid in mucus was Lys, in foot tissues—Trp and in shells—His. The concentrations of Ile, Thr and Val in snail tissues were dominating in comparison with this reference pattern. CS for amino acids in mucus and foot tissues were <1.00, which showed that their amounts were smaller comparing to whole egg protein reference pattern. According to the CS, the first limiting amino acids in mucus were Trp and Met + Cys and in foot tissues and shells—Met + Cys. Met content in mucus was very low comparing to other amino acids and its amount in other tissues.

Cavuoto and Fenech demonstrated that Met restriction in human diet may be major strategy of control of cancer growth, particularly in cancers exhibiting Met dependence for survival and proliferation [25]. Restriction of Met resulted in selective killing of cancer cells, which depend on Met, in co-culture with non-cancer cells. Several studies on animals, in which Met-restricted diet was tested, reported cancer growth inhibition and healthy life-span extension. Met depletion, in cancer cells which depend on Met, can lead to cell cycle arrest in late S/G_2_ phase, in vitro and in vivo. Cells which are arrested in late S/G_2_ phase are susceptible to death and hypersensitive to chemotherapy.

Among NEAA, Glu, Asp and Gly were the most abundant in snail tissues.

The study in Nature demonstrated that deprivation of dietary serine (Ser) and Gly inhibits growth of some tumors, including intestinal cancer [26,27]. Whether this intervention is successful depends on the oncogenic context and origin of tumor tissue. Dietary Gly depletion limits one route of synthesis of Ser and both Ser and Gly removal from the diet leads to their lower levels in plasma. Deprivation of Ser and Gly may decrease tumor ability to cope with reactive oxygen species (ROS). Moreover, combination of ROS-inducing treatment, as radiation, with deprivation of Ser and Gly may prove effective. Deprivation of Ser and Gly limits one-carbon units for biosynthesis of nucleotides and this intervention might empower efficacy of drugs targeting nucleotide synthesis.

Some individual amino acids exhibited the potential to attenuation of carcinogensis mainly by angiogenesis inhibition [28]. The example is the activity of arginine (Arg), presented in all examined snail tissue lyophilisates, in SW480 human colon cancer cells and in SW480 colon cancer cells xenograft mouse model. In turn, Gly inhibited tumor growth in model animals. Moreover, administration to obese diabetic rats of branched-chain amino acids (Leu, Ile, Val) indicated their ability to attenuation of development of preneoplastic lesions and inhibition of angiogenesis.

EAA/TAA and EAA/NEAA ratios for examined snail tissues were similar to the reference values of FAO/WHO [24]. In the work of Bonfili et al., two mixtures of amino acids, first consisting of EAA and a second consisting of 85% EAA and 15% NEAA, were examined to explore their influence on viability of epithelial cells, MCF 10A and cancer cells (human colon carcinoma HCT 116, cervical adenocarcinoma HeLa, human hepatocellular carcinoma Hep G2, MCF7 and Caco-2), and to clarify involved molecular mechanisms [23]. Both mixtures showed cell-dependent, antiproliferative and cytotoxic effects, involving proteasome activity inhibition and consequently autophagy and apoptosis activation. The results exhibited that different ratios of EAA and NEAA can influence survival of cancer cells. Thus, customization by specific amino acid mixtures of ratios among EAA and NEAA may represent an anticancer strategy, which is able to selectively cause death of cancer cells. In turn, administration of EAA, with or without doxorubicin, a cancer chemotherapy agent, increased mortality of the cancer cell lines: HCT 116, MCF7 and M14 (melanoma) [29]. EAA alone caused an increase in the apoptotic markers, cleaved Caspase-3 and Bax. Therefore, a “positive imbalance” between EAA and NEAA may alter the environment of cancer cells and trigger a signaling cascade inducing the events which are not compatible with survival and proliferation of cancer cells. 

Snail foot tissues contained slightly less PUFA, including TFA, compared to shells. In turn, concentration of MUFA in foot tissue was lower and of SFA higher than in shells. According to FAO, the SFA total intake should not be higher than 10% energy (E) [22]. The MUFA intake depends on intake of total fat and fatty acid pattern and may cover a wide range.

In shells, we quantified myristic acid, a medium chain fatty acid with proven antiproliferative properties [30]. Fauser et al. demonstrated that pre-treatment of liposomes with myristic acid delivered to mice bearing B16 melanoma tumors delayed tumor initation and death.

Our study revealed higher concentration of n-3 PUFA (sum of α-linolenic acid, eicosatrienoic acid and EPA) in foot tissues than in shells, however, the content of α-linolenic acid was higher in shells and foot tissues were much richer in EPA. According to FAO, the total intake of n-3 fatty acids can range between 0.5 and 2% E and the minimum dietary requirement for α-linolenic acid (>0.5% E) protects from symptoms of deficiency in adults [22].

The content of n-6 PUFA turned out to be higher in shells. The concentrations of linoleic and γ-linolenic acids were higher in shells, of eicosadienoic and dihomo-γ-linolenic—in foot tissues. Docosadienoic acid was presented only in foot tissues. According to FAO, the acceptable macronutrient distibution range (AMDR) for intake of n-6 fatty acids (linoleic acid) is 2.5–9% E [22].

Foot tissues of snails had a balanced n-6/n-3 fatty acid ratio. It was demonstrated that high n-6/n-3 ratio is connected with increase of inflammation and consequently cancer development [14]. However, in population-based study was not found any evidence that n-6 PUFA increase and n-6/n-3 ratio decreases colorectal cancer risk [31]. The results of that study suggest that intake of n-3 PUFA of marine origin may be related inversely to the risk of cancer in the proximal site of large intestine. Bathen et al. proved that modification of diet with fish oil, rich in n-3 PUFA, influence on suppression of growth of colon cancer xenografts in nude mice [2]. In addition to experimental results, human studies support suppressive effects of n-3 PUFA against initiation [32] and progression [33] of colorectal tumor cells.

In turn, Zhang et al. demonstrated that viability of colon cancer cells, LoVo and RKO, was suppressed by α-linolenic, linoleic, EPA and γ-linolenic acids in a dose- and time-dependent manner [34]. RKO, as semi-differentiated cells, were more sensitive than LoVo (undifferentiated cells). Tested fatty acids were capable of inducing apoptosis and EPA and γ-linolenic acid were the most effective. Apoptosis was mediated through mitochondria-mediated pathway which was evidenced by mitochondrial membrane potential loss, ROS generation, intracellular Ca^2+^ accumulation, Caspases-9 and -3 activation, ATP level decrease and Bax/Bcl-2 expression ratio increase.

In the study of Yang et al., treatment the spheres from SW620 cell line, colorectal cancer stem-like cells, with n-3 EPA and DHA (docosahexaenoic acid), alone or in combination, induced apoptosis, loss of viability, DNA fragmentation and increase in expression of annexin V [35]. Moreover, it was observed that EPA and DHA, especially EPA combined with DHA, can enhance the effect of chemotherapeutic sensitivity to fluorouracil and mitomycin C.

The human P-glycoprotein (P-gp), coded by multidrug resistance (MDR1) gene, is an ATP-dependent membrane efflux pump which decreases intracellular retention of anticancer drugs and is related to multidrug resistance [36]. EPA, DHA and arachidonic acid, in different concentrations, can cause reduction in gene expression and the production of protein and pump activity of MDR1 in Caco-2 cells. Moreover, incubation with these PUFA enhanced cytotoxicity of paclitaxel, anticancer drug, manifested mainly through induction of apoptosis.

Cai et al. demonstrated that DHA, and to a lesser extent EPA, decreased viability and increased apoptosis and production of peroxides after radiation therapy in colorectal cancer LS174T cells and to a lesser extent in less sensitive to radiation therapy (HT-29 cells) [3]. Higher expression of heat shock protein 70, lower expression of NF-κB p65, COX-2 and Bcl-2 were noted. The effect was synergistic in LS174T cells and additive in HT-29 cells. Enhanced cytotoxicity was at least partly due to lipid peroxidation and consequently modulation of inflammatory response and induction of apoptosis.

Supplementation with n-3 PUFA leaded to reduction of cell proliferation and increase of apoptosis in crypts of patients with colorectal cancer history [14]. Thus, n-3 PUFA can be used as a targeted treatment in combination with radiotherapy or chemotherapy of colorectal cancer. 

Both snail tissues comprised little TFA, which intake should be no more than 1% E [22]. 

The content of minerals in a diet is highly important and their role results not only from nutritional value and physiological functions. Mineral compounds influence on taste of food, they are catalyzers and inhibitors of many reactions.

Na content in lyophilised snail foot tissues was about four times higher than in dry matter of edible parts of oysters [37] or pork meat [38]. However, WHO recommends reduction of Na consumption to <2 g/day in adults [39].

Lyophilisates of examined snail tissues were multiple times richer in Ca comparing to dry matter of lean beef [18], pork [38] or edible parts of oysters [37]. In turn, P concentration in beef [18], pork meat [38] and oysters [37] was similar to snail foot tissues. Zhang and Giovannucci showed that Ca has anti-colorectal cancer properties including stimulation of differentiation, reduction of proliferation and induction of apoptosis [40]. Moreover, Ca and P exhibited a protective action at certain steps of adenoma-carcinoma sequence [41].

WHO suggests K intake of at least 3.51 g/day for adults, which corresponds to 352 g of snail tissue lyophilisate, in which K content is similar to the content of this element in dry matter of oysters edible parts [37,42]. However, there was no significant association between dietary intake of K and colorectal cancer risk [43].

Recommended nutrient intake (RNI) for Mg is 220 mg/day for females (19–65 years) and 260 mg/day for males [44]. 220 mg was contained in 39 g of lyophilised snail mucus, 145 g of foot tissues and 344 g of shells. Mg concentration in mucus and foot tissues was higher comparing to dry matter of beef [18], pork meat [38] and oysters [37]. Higher intake of Mg seemed to be connected with a modest decrease of colorectal cancer risk, in particular colon cancer [45].

Lyophilised foot tissues and shells of *H. aspersa* Müller were rich in S. One study indicated apoptosis-inducing capability of S in drug-resistant non-small cell lung carcinoma (NSCLC) cells, by shifting cellular environment from NF-κB-mediated survival milieu to p53-mediated apoptosis [46]. S is also a component of non-enzymatic antioxidants being therapeutic tools for cancer [47].

Examined snail tissues, especially mucus, had higher levels of Cu than beef [18], pork meat [38] and oysters edible parts [37]. In the study of Davis and Johnson, dietary Cu did not influence on colon tumor incidence in rats [48]. However, Cu induced cytotoxicity in human colon cancer cells HT-29, which was related to induction of apoptosis, increase in oxidative stress, changes in mitochondrial β-oxidation and configuration of lipid and energy metabolism [49]. Moreover, Cu exerted intracellular, toxicological influence on Caco-2 cells and the effect seemed to be more evident in post-confluent than pre-confluent cells [50]. In turn, rats which administered low dietary Cu had 105% higher induced preneoplastic lesions of colon cancer than animals fed adequate dietary Cu [51].

Mn content in lyophilised snail tissues, examined in the present study, was several times higher than in dry matter of edible parts of oysters [37]. The study of Davis and Feng showed that the rats fed low dietary Mn had 23% higher preneoplastic lesions and animals ingesting high dietary Fe had 18% higher preneoplastic lesions [51]. Generally, approximately three-quarters of studies assessing relation between Fe intake and colorectal cancer associated higher Fe intake with increased risk of colorectal cancer [52]. Fe-enriched diets were similarly shown to increase incidence of tumors in a mouse model of colitis. Moreover, high-Fe diet enhanced proliferation and large adenoma formation in azoxymethane-induced mouse colon cancer model. In turn, low-Fe diets diminished colon cancer xenografts growth in mice. Fonseca-Nunes et al. showed that Fe may influence colorectal carcinogenesis by formation of ROS, leading to DNA damage [53]. Heme Fe may play the most important function in colorectal carcinogenesis. Heme Fe catalyzes formation of N-nitroso compounds and end products of lipid peroxidation. Senesse et al. demonstrated that Fe loading to SW480 and Caco-2 cells caused proliferation and repression of E-cadherin [54]. Progression of colorectal cancer is related to higher expression of Fe import proteins and a block in Fe export, which results in higher levels of intracellular Fe, that may enhance proliferation and repress adhesion of cells. According to FAO/WHO, RNI for Fe depends from its bioavailability and ranges from 9.1 to 27.4 mg/day (females, 18+) and from 19.6 to 58.8 mg/day (males, 18+) [44]. 9.1 mg was contained in 323 g of lyophilised snail mucus, 90 g of foot tissues and only 12 g of shells. Fe concentration in shells was several times higher than in dry matter of beef [18], pork meat [38] and edible parts of oysters [37]. 

RNI for dietary Zn depends on its bioavailability and ranges from 3.0 to 9.8 mg/day (females, 19–65 years) and from 4.2 to 14.0 mg/day (males, 19–65 years) [44]; 3 mg was contained in 57 g of lyophilised snail mucus, 46 g of foot tissues and 139 g of shells. Zn concentration in lyophilised snail tissues, investigated in the present study, was several times higher than in dry matter of pork meat [38], but lower than in beef [18] and edible parts of oysters [37]. Li et al. showed that dietary Zn intake was inversely connected with cancers of digestive tract, especially with risk of colorectal cancer [55].

In turn, administration of Zn to dimethylhydrazine-treated rats decreased the incidence of tumors, their multipilicity and size [56]. Moreover, increased Zn levels inhibited growth and induced death of cell lines which represent different colon cancer stages: HT-29, HCT-116 and SW620 [57]. The most important mechanism was oxidative stress which activated stress kinase-dependent signaling, mitochondria perturbation and damage of plasma membrane. Cell death depended on cell line and was variable; cells displayed features of apoptosis, autophagy, necrosis and other mixed-types.

B concentration in lyophilised snail tissues was higher than in dry matter of edible parts of oysters [37]. Work of Brookes et al. presented that B is associated with lower risk of different types of cancer [58].

The incidence of esophageal tumors induced by *N*-methyl-*N*-benzylnitrosamine and their development were significantly diminished in rats on high-Mo diet comparing to rats on low-Mo diet [59]. The results indicated that xanthine oxidase plays a significant role in inhibition of esophageal carcinogenesis by Mo. In our study, lyophilised mucus was richer in Mo than other tissues.

Cr content in *H. aspersa* Müller foot tissue lyophilisate was ten times higher comparing to the content in dry matter of oysters edible parts [37]. In the study of Odukanmi et al., trivalent Cr improved colitis healing in mice by promotion of antioxidant activities, suppression of ROS and inflammation [60]. In turn, chronic administration of hexavalent Cr in high doses elicited alimentary cancers in mouse models [61]. Differentially expressed genes analyses demonstrated over-represented functions connected with oxidative stress, lipid metabolism, immune responses and cell cycle, consistent with the reported influence on redox state and histopathology.

Epidemiological studies indicated close correlation between Ni exposure and lung cancer incidence [62]. Moreover, several studies suggested that Ni contributes to progression of human lung cancer. Ni content in snail tissue lyophilisates did not considerably differ from its content in dry matter of edible parts of oysters [37]. 

RNI for Se is 26 μg/day for females (19–65 years) and 34 μg/day for males [44]; 26 μg was contained in 96 g of lyophilised snail mucus and 72 g of foot tissues. Se content in lyophilised snail tissues was a little bit lower than in dry matter of beef [18] and edible parts of oysters [37]. In the work of Nolfo et al., the potential prevention of colorectal cancer by Se was attributed to its functions in acceleration of DNA repair, reduction of DNA damage and decrease in mutations contributing to carcinogenesis [63]. Another mechanism could be related to induction of apoptosis, by the activation of Caspase-3 and modulation of glutathione and functions of mitochondria. In turn, after porcine sera biofortified with Se was added to HCT 116 cells, prostate cancer cells DU 145 and small cell lung cancer cells NCI-H446, it decreased their viability by promoting apoptosis [64]. This influence was replicated only by the addition to serum of methylseleninic acid and was mediated by downregulation of cell cycle arrest genes and upregulation of apoptotic genes. Selenoproteins H, K, M, N and T turned out to be strongly connected with signaling related to cell death induced by porcine serum enriched in Se.

Treatment with extracts from mucus for 72 h resulted in a decrease of viability of Caco-2 cells, up to 76% of viability of control. Only molecules from fraction <50 kDa turned out to reduce viability. The effect of mucus against Caco-2 cells could be assigned to the presence of proteins and peptides demonstrating anticancer properties, a little amount of Met, adequate ratio of EAA to NEAA and such elements as Cu, Ca, Zn and Se, and their interactions. Treatment with extract from foot tissues in a concentration of 2500 μg/mL decreased viability of Caco-2 cells and the effect was visibile after 24 h. The particles from fractions of molecular weights <50 kDa, <10 kDa and <3 kDa were revealed to be responsible for reduction of viability. The anticancer action of foot tissues, revealed in our study, could be related to the presence of components contained in mucus, as well as products of lipid peroxidation and polyunsaturated fatty acids, such as n-3—EPA and α-linolenic acid, and n-6-linoleic and γ-linolenic acids, and their interactions. Molecules of different molecular weights from shells increased cell viability after 24 h. One of them could be Fe.

## 4. Materials and Methods

### 4.1. Animal Material and Preparation of Samples

*Helix aspersa* Müller, 1774 ((*Cornu aspersum* (Müller, 1774)), known by the common name garden snail, live samples used in the present experiments were obtained from the commercial breeding in the Łódź area (Łódź, Poland). Snails, with the initial body weight of 11–15 g (*n* = 100), were kept in the plastic box with the ventilation openings, under proper zoohygienic conditions. Every other day the box was washed and fresh standard diet was provided. The animals had free access to water.

To collect the crude mucus, snails were treated for a fraction of second by an electric impulse, which were generated from the current source of 4.5 V, and then their foot muscles were stimulated. After mucus collection snails were left for regeneration. From the regenerated animals foot tissues (with head) were prepared and shells were taken and rinsed in running water. Harvested mucus, foot tissues and shells derived from all snails were homogenized, frozen at −80 °C, lyophilised (Lyovac GT 2 freeze-dryer, SRK Systemtechnik GmbH, Riedstadt, Germany), milled into fine powder, separated for individual analyses and stored at −80 °C to the moment of experiments. 

### 4.2. Red-Ox State Indicators

#### 4.2.1. Ferric-Reducing Antioxidant Power

Ferric reducing antioxidant power of lyophilised mucus, foot tissues and shells of *H. aspersa* Müller was assayed according to the modified method of Oyaizu [65,66]. The method is based on reduction of Fe^3+^ being in stoichiometric excess comparing to the antioxidants, due to electron donation by these compounds, the absorbance increases with higher reduction capability. Tissue extracts were obtained after homogenization of lyophilisates in phosphate-buffered saline (PBS) and centrifugation (1600 *g*, 10 min). Extracts (2.5 mL) were mixed with sodium phosphate buffer (2.5 mL, 0.2 M, pH 6.6) and potassium ferricyanide (2.5 mL, 1%). The mixture was incubated at 50 °C (20 min) and then trichloroacetic acid (TCA, 2.5 mL, 10%) was added. After centrifugation (3000× *g,* 5 min), the supernatant (0.4 mL) was mixed with deionized water (0.4 mL) and ferric chloride (160 μL, 0.1%), and the absorbance was read at 700 nm, applying microplate reader (Infinite M200, Tecan, Männedorf, Switzerland). The standard curve was prepared using different concentrations, ranging 0–100 µM, of (±)-6-hydroxy-2,5,7,8-tetramethylchromane-2-carboxylic acid (TROLOX), a water-soluble analog of vitamin E.

#### 4.2.2. Scavenging Activity of 2.2′-Azino-bis(3-ethylbenzthiazoline-6-sulfonic Acid) Radical Cation (ABTS*^·^*^+^)

ABTS*^·^*^+^ is a relatively stable free radical, decolorizing when reduced. To assess the ABTS^·+^ scavenging activity of extracts from *H. aspersa* Müller tissues (as in Section 4.2.1), the modified procedure of Sun et al. was used [66]. In this method, antioxidants are added to ABTS^·+^ solution and remaining ABTS*^·^*^+^ is determined spectrophotometrically. The ABTS reagent was prepared by combining 5 mL of 7 mM ABTS with 88 μL of 140 mM K_2_S_2_O_8_. To allow the radical generation, the mixture was placed in the dark, at room temperature, for 16 h. Then, the reagent was diluted with 99.8% ethanol, to adjust the absorbance to 0.70 ± 0.02 at 734 nm (microplate reader Infinite M200, Tecan, Männedorf, Switzerland). To assay the radical cation scavenging activity, 0.9 mL of ABTS reagent was mixed with 0.1 mL of snail tissue extracts and after 6 min of incubation, at room temperature, the absorbance was monitored. The standard curve was prepared by applying TROLOX, as in Section 4.2.1.

#### 4.2.3. Scavenging Activity of 2.2-Diphenyl-1-picrylhydrazyl Radical (DPPH^·^)

To determine the DPPH^·^ scavenging activity of snail tissue extracts (as in Section 4.2.1), the modified method described by Li et al. was utilized [67]. The method is based on ability of donation by antioxidants of hydrogen atom or electron to the odd electron in DPPH^·^, the absorbance decreases proportionally to rise in non-radical form of DPPH. DPPH^·^ solution (0.2 mM) in absolute methanol was mixed vigorously with snail extracts (2:1, *v*/*v*). After 30 min of incubation, without access to light, the mixture was centrifuged (15,000× *g*, 10 min) and the absorbance of the supernatant was recorded at 517 nm, using microplate reader (Infinite M200, Tecan, Männedorf, Switzerland). The standard curve was prepared as in Section 4.2.1.

#### 4.2.4. Peroxidation of Lipids

Lipid peroxidation products were evaluated in extracts from examined tissues of *H. aspersa* Müller as thiobarbituric acid reactive substances (TBARS), according to the method of Uchiyama and Mihara [68]. Extracts were obtained after homogenization of tissue lyophilisates in Radio-Immunoprecipitation Assay (RIPA) Buffer and centrifugation (1600× *g* 10 min). TBARS were expressed as malondialdehyde (MDA) equivalents, and the precursor of MDA—1.2.3.3-tetraethoxypropane (TEP)—was utilized as a standard. The absorbance was measured at 532 nm, applying microplate reader (Infinite M200, Tecan, Männedorf, Switzerland). 

### 4.3. Proximate Composition

The representative samples of mucus, foot tissues and shells were analyzed for contents of dry matter, crude protein, crude fat and crude ash, according to AOAC International [69]. Crude fat was extracted using petroleum ether by the Soxhlet method.

### 4.4. Analysis of Molecular Weights of Proteins

To prepare the protein extracts, the snail tissue lyophilisates were homogenized in PBS with protease and phosphatase inhibitors (Sigma-Aldrich, St. Louis, MO, USA) and centrifuged (1600× *g*, 10 min). Total protein concentrations in extracts were determined by the Bradford method with bovine serum albumin (BSA) as the standard [70]. Then, samples with equalized concentrations of total protein were subjected to sodium dodecyl sulfate-polyacrylamide gel electrophoresis (SDS-PAGE), using a 5% stacking gel and 10% resolving gel, according to Laemmli method [71] with modifications. Briefly, 15 µL of samples were denatured and reduced with 15 µL of Laemmli Sample Buffer with β-mercaptoethanol (Bio-Rad Laboratories, Hercules, CA, USA) and heating (95 °C, 5 min). Then, 20 µL of each of them and 5 µL of protein marker (ColorBurst™ Electrophoresis Marker, Sigma-Aldrich, St. Louis, MO, USA) were loaded onto the gel and resolved, applying the Mini-PROTEAN^®^ electrophoresis system (Bio-Rad Laboratories, Hercules, CA, USA). The protein bands, separated on gel, were fixed, stained in QC Colloidal Coomassie Stain and destained according to the producer procedure (Bio-Rad Laboratories, Hercules, CA, USA). Then, the gel was visualized using GelDoc Imaging System (Bio-Rad Laboratories, Hercules, CA, USA). 

### 4.5. Analysis of Amino Acids

The contents of all of the amino acids except tryptophan (Trp) in lyophilised mucus, foot tissues and shells of *H. aspersa* Müller were determined by ultraperformance liquid chromatography with photodiode array detection (UPLC-PDA). The analysis was performed by applying Acquity UPLC System with PDA detector, wavelength 260 nm (Waters Corp., Milford, MA, USA) and AccQ-Tag Ultra C18, 1.7 µm, 2.1 × 100 mm column (Waters Corp., Milford, MA, USA). The detailed information concerning validation of the method can be found elsewhere [72]. 

The content of Trp was evaluated by high-performance liquid chromatography with fluorescence detection (HPLC-FLD, Agilent 1100 Series, Agilent Technologies, Santa Clara, CA, USA) [73]. Zorbax^®^ ODS C18, 4.6 mm ID × 250 mm (5 μm) column (Agilent Technologies, Santa Clara, CA, USA) was applied.

Analysis of amino acids was performed in the laboratory which is accredited by the Polish Centre for Accreditation.

The amino acid score (AAS), chemical score (CS) and essential amino acid index (EAAI) were computed by applying the following equations [24,74]:

$$\mathrm{AAS}=\;\frac{\mathrm{aa}}{\mathrm{AA}\;\left(\mathrm{standards}\right)}$$

$$\mathrm{CS}=\;\frac{\mathrm{aa}}{\mathrm{AA}\;\left(\mathrm{Egg}\right)}$$

$$\mathrm{EAAI}=\;\sqrt[{n}]{\frac{100A}{\mathrm{AS}}\;\times \;\frac{100B}{\mathrm{BS}}\;\times \;\frac{100C}{\mathrm{CS}}\;\times \;\ldots \;\times \frac{100H}{\mathrm{HS}}}$$

where aa is the amount of amino acid per test protein (%); AA (standards) is the amount of amino acid per protein in FAO (the Food and Agriculture Organization of the United Nations)/WHO (the World Health Organization) reference pattern for preschool children, 2–5 years (%), AA (Egg) is the amount of amino acid per protein in whole egg protein reference pattern (%); n is the number of amino acids; A, B, C, …, H is the amount of essential amino acids per test protein (%); AS, BS, CS, …, HS is the amount of essential amino acids per protein in reference pattern (%).

### 4.6. Composition of Fatty Acids

The analysis of fatty acid methyl esters (FAME) was performed using gas chromatograph (Agilent 7890A, Agilent Technologies, Santa Clara, CA, USA) with the capillary column: 105 m length, 0.25 mm diameter, 0.2 μm df (Restek Rtx-2330, Restek Corp., Bellefonte, PA, USA), with the photoionization detector (FID), in the laboratory which is accredited by the Polish Centre for Accreditation [75]. FAME in the analyzed samples were identified by comparison of their retention times with retention times of individual standards and expressed in g per 100 g of total amount of FAME. The calibration was conducted by applying the mixture of the certified reference material FAME Mix, C4-C24 (Sigma-Aldrich, St. Louis, MO, USA), the single fatty acid standards and the certified reference material soya maize oil blend (fatty acid profile, Sigma-Aldrich, St. Louis, MO, USA). C18:2 and C18:3 *trans* isomers were identified based on the last reference material.

### 4.7. Analysis of Minerals

The concentrations of Na, Ca, K, Mg, P, Cu, Zn, Fe, Mn in mucus, foot tissues and shells of snails were determined by inductively coupled plasma—atomic emission spectroscopy (ICP-AES, iCAP 6500, Thermo Scientific, Waltham, MA, USA) [76]. The contents of B, Mo, Cr, Ni, Se, Co in above tissues were evaluated by mass spectrometry with ionization in inductively coupled plasma (ICP-MS, Varian 820-MS, Varian, Inc., Palo Alto, CA, USA) [77]. The levels of S, Cl, Si in foot tissues and shells were measured by wavelength-dispersive X-ray fluorescence (WDXRF, Axios, PANalytical, Almelo, The Netherlands) [78]. The analysis was performed in the laboratories which are accredited by the Polish Centre for Accreditation. 

### 4.8. Preparation and Fractionation of Extracts for Cell Viability Tests

For cell viability tests, the lyophilised mucus, foot tissues and shells of snails were homogenized in deionized water and centrifuged (1600× *g*, 10 min). Then, the supernatants were filtered using polyvinylidene fluoride (PVDF) syringe filters with pore size 0.22 µm (EuroClone, Pero, Italy).

Part of the extracts was fractionated, based on the molecular size, using ultra centrifugal filter devices with regenerated cellulose membrane (Merck Millipore, Burlington, MA, USA), with 3, 10 and 50 kDa cutoffs, according to the producer recommendations in regard to spin time and g-force. Six fractions were obtained: >50 kDa (50K), 10–50 kDa (10K), 3–10 kDa (3K), <50 kDa (50F), <10 kDa (10F) and <3 kDa (3F). 

All of the extracts and fractions were sterilized using syringe filters with pore size 0.22 μm (EuroClone, Pero, Italy) under the biological safety cabinet (class II, Esco Technologies, Inc., Horsham, PA, USA), before viability tests. 

### 4.9. Caco-2 Cell Culture

Human epithelial colorectal adenocarcinoma (Caco-2) cell line (ECCC, 55 passage; Sigma-Aldrich, St. Louis, MO, USA) was grown in 96-well plastic plates (at a density of 1 × 10^4^ cells/100 µL) in Minimum Essential Medium (MEM) containing 2 mM l-glutamine (Thermo Fisher Scientific, Waltham, MA, USA), with 10% fetal bovine serum (FBS; Thermo Fisher Scientific, Waltham, MA, USA), 1% non-essential amino acids (NEAA, Thermo Fisher Scientific, Waltham, MA, USA) and 1% antibiotic-antimycotic (Thermo Fisher Scientific, Waltham, MA, USA) [79]. The cultures were kept at 37 °C in 5% CO_2_ and 95% relative humidity (CO_2_ incubator INCO 108 med, Memmert GmbH + Co. KG, Schwabach, Germany). After 24 h of incubation and reaching 70% confluence, the cells were starved overnight in MEM (Thermo Fisher Scientific, Waltham, MA, USA) with 1% FBS (Thermo Fisher Scientific, Waltham, MA, USA) and 1% antibiotic-antimycotic (Thermo Fisher Scientific, Waltham, MA, USA).

### 4.10. Influence of Extracts on Cell Viability

To Caco-2 cells were added 90 μL of new culture medium and 10 μL of extracts from mucus, foot tissues and shells of snails, at the concentrations of 2500, 250, 25 and 2.5 μg/mL. Equal volumes of sterile deionized water were added to the control cells. Different additional controls were also applied. After 24 and 72 h of incubation (37 °C, 5% CO_2_ and 95% relative humidity, incubator INCO 108 med, Memmert GmbH + Co. KG, Schwabach, Germany), the MTT test was performed according to the modified method of Tada et al. [80]. In brief, 15 μL of 5 mg MTT reagent (methylthiazolyldiphenyl-tetrazolium bromide, Sigma-Aldrich, St. Louis, MO, USA)/mL PBS was added to each well and plates were incubated at 37 °C for 4 h. Then, 100 μL of lysis buffer (10% SDS in 0.01 N HCl) was added, plates were incubated at 37 °C over night and the absorbance was read at 570 nm (microplate reader Infinite M200, Tecan, Männedorf, Switzerland).

### 4.11. Influence of Fractions of Extracts on Cell Viability

To Caco-2 cells were added 90 µL of new culture medium and 10 µL of six fractions: >50 kDa (50K), 10–50 kDa (10K), 3–10 kDa (3K), <50 kDa (50F), <10 kDa (10F) and <3 kDa (3F) of extracts from mucus, foot tissues and shells of snails, at the concentrations of 25 and 2.5 µg/mL. The same controls were applied as in Section 4.10. After cells incubation with the fractions of extracts from foot tissues and shells for 24 h and fractions of extract from mucus for 72 h, the MTT test was performed, as in Section 4.10.

### 4.12. Statistical Analysis

The results were expressed as the mean values ± the standard error of the mean (SEM). One-way analysis of variance (ANOVA) was applied and mean values were compared using the Tukey’s post-hoc test. The differences at *p* <0.05 were considered statistically significant. Statistical analysis was performed with Statgraphics Centurion software (StatPoint Technologies, Inc., Warrenton, VA, USA). 

## 5. Conclusions

*H. aspersa* Müller tissue lyophilisates contained antioxidants which were characterized by electron or hydrogen radical donor properties for neutralizing free radicals, that could be important especially for oxidative stress decrease in prevention and preneoplastic state of colorectal cancer. We confirmed the presence in snail tissue lyophilisates of a wide array of compounds that might be used in treatment of colorectal cancer. The decrease of viability of the colon cancer cell line, Caco-2, after the application of extracts from lyophilized mucus and foot tissues of *H. aspersa* Müller was affirmed in our study for the first time. The anticancer effect of extract from mucus on the Caco-2 cell line could be related to the content of some proteins and peptides, proper EAA/NEAA ratio, Met restriction and the presence of some minerals, such as Cu, Ca, Zn, Se. The influence of extract from foot tissues against Caco-2 cells could be assigned additionally to the presence of EPA, α-linolenic, linoleic and γ-linolenic acids. The opposite effect on Caco-2 cells was demonstrated by extract from lyophilized shells which increased their viability, suggesting effects that are different from cancer treatment. Further studies are needed to know whether dietary intervention by *H. aspersa* Müller mucus and foot tissues would form an effective approach in colorectal cancer management. The most subsequent step in such studies seems to be elucidation of mechanisms of anticancer effects of extracts from *H. aspersa* Müller tissues. Important factors could be the influence of extracts on factors such as level of oxidative stress, activation of apoptosis and apoptotic proteins and the cell cycle. There is also more need for further identification of bioactive compounds of mucus and foot tissues of *H. aspersa* Müller with potential anticancer action. 

## Figures and Tables

**Table 1:** Proximate composition of different lyophilised tissues of *Helix aspersa* Müller.

Compound (% of Lyophilisate)	Mucus	Foot Tissues	Shells
Crude protein	55.84	80.74	1.98
Crude fat	0.35	3.67	0.17
Crude ash	30.60	8.03	91.43

**Table 2:** Amino acid composition of different lyophilised tissues of *Helix aspersa* Müller (mg/g crude protein) ^1^.

Amino Acids	Mucus	Foot Tissues	Shells
	Essential Amino Acids (EAA) *		
Isoleucine	37.82 ± 0.02	36.11 ± 0.03	109.96 ± 1.01
Leucine	75.42 ± 0.28	62.03 ± 0.17	164.41 ± 1.31
Lysine	40.79 ± 1.32	53.10 ± 2.31	129.76 ± 3.13
Methionine	4.05 ± 0.06	14.28 ± 0.24	33.92 ± 0.56
Phenylalanine	40.46 ± 1.31	34.23 ± 1.35	106.81 ± 0.08
Threonine	44.29 ± 0.18	39.41 ± 0.37	106.38 ± 1.77
Valine	45.05 ± 0.19	40.36 ± 0.28	160.57 ± 2.68
Tryptophan	8.87 ± 0.12	7.88 ± 0.17	22.38 ± 1.06
	Half-Essential Amino Acids (HEAA) *		
Arginine	40.84 ± 0.85	70.95 ± 1.91	126.66 ± 0.79
Histidine	18.08 ± 0.59	15.54 ± 0.29	32.86 ± 1.29
	Non-Essential Amino Acids (NEAA) *		
Cysteine	25.79 ± 0.57	10.31 ± 0.21	27.66 ± 0.17
Aspartic acid ^#^	87.80 ± 1.52	89.03 ± 1.70	231.24 ± 3.44
Glycine ^#^	63.75 ± 0.71	64.04 ± 1.73	225.28 ± 1.57
Glutamic acid ^#^	82.74 ± 0.69	132.56 ± 2.24	271.57 ± 3.46
Alanine ^#^	33.67 ± 0.39	46.61 ± 0.47	117.79 ± 1.21
Serine	42.37 ± 0.23	47.90 ± 0.66	119.31 ± 3.74
Proline	32.80 ± 0.10	41.41 ± 0.14	93.50 ± 0.46
Tyrosine	33.85 ± 1.40	30.86 ± 1.36	50.89 ± 1.64
	**Amino Acid Groups and Ratios**		
Total amino acids (TAA)	758.42 ± 2.32	836.56 ± 0.00	2103.24 ± 24.37
Essential amino acids (EAA)	296.73 ± 0.48	287.37 ± 1.46	834.18 ± 6.80
Half-essential amino acids (HEAA)	58.92 ± 1.44	86.49 ± 2.19	159.51 ± 2.07
Non-essential amino acids (NEAA)	402.77 ± 0.40	462.70 ± 0.73	1109.55 ± 15.51
Delicious amino acids (DAA)	267.97 ± 1.90	332.23 ± 2.68	845.87 ± 9.68
EAA/TAA	0.39	0.34	0.40
EAA/NEAA	0.74	0.62	0.75
DAA/TAA	0.35	0.40	0.40

^1^ Data are expressed as mean ± standard error of the mean (SEM); * for humans, ^#^ delicious amino acids. *n* = 2.

**Table 3:** Amino acid score (AAS), chemical score (CS) and essential amino acid index (EAAI) of mucus, foot tissues and shells of *Helix aspersa* Müller ^1^.

Amino Acids	AAS			CS		
	Mucus	Foot Tissues	Shells	Mucus	Foot Tissues	Shells
Histidine	0.95	0.82	1.73	0.82	0.71	1.49
Isoleucine	1.35	1.29	3.93	0.70	0.67	2.04
Leucine	1.14	0.94	2.49	0.88	0.72	1.91
Lysine	0.70	0.92	2.24	0.58	0.76	1.85
Threonine	1.30	1.16	3.13	0.94	0.84	2.26
Tryptophan	0.81	0.72	2.03	0.52	0.46	1.32
Valine	1.29	1.15	4.59	0.68	0.61	2.43
Methionine + cysteine	1.19	0.98	2.46	0.52	0.43	1.08
Phenylalanine + tyrosine	1.18	1.03	2.50	0.80	0.70	1.70
EAAI	107.80	98.63	266.65	70.18	64.21	173.59

^1^ Grey fields—the first limiting amino acids.

**Table 4:** Comparison of composition of fatty acids in foot tissues and shells of *Helix aspersa* Müller ^1^.

Fatty Acids (g/100 g FAME)	Foot Tissues	Shells	*p*
C14:0 (myristic)	-	0.227 ± 0.004	
C15:0 (pentadecanoic)	-	0.201 ± 0.009	
C16:0 (palmitic)	5.02 ± 0.33	5.20 ± 0.40	0.7457
C16:1 *cis*-9 (palmitoleic)	0.059 ± 0.011 ^a^	0.253 ± 0.047 ^b^	0.0154
C17:0 (margaric)	1.317 ± 0.073 ^B^	0.243 ± 0.010 ^A^	0.0001
C18:0 (stearic)	16.27 ± 0.03 ^B^	4.71 ± 0.22 ^A^	0.0000
C18:1 *cis*-9 (oleic)	11.6 ± 0.2 ^A^	30.0 ± 1.3 ^B^	0.0002
C18:1 *cis*-11 (*cis*-vaccenic)	0.416 ± 0.018 ^A^	1.337 ± 0.087 ^B^	0.0005
C18:2 all *trans*-9,12 (linolelaidic)	0.198 ± 0.039 ^b^	0.076 ± 0.003 ^a^	0.0356
C18:2 *trans* isomer	0.147 ± 0.000 ^B^	0.099 ± 0.006 ^A^	0.0010
C18:2 all *cis*-9,12 (linoleic), n-6	16.4 ± 0.5 ^A^	28.1 ± 0.6 ^B^	0.0001
C18:3 all *cis*-6,9,12 (γ-linolenic), n-6	0.091 ± 0.018	0.101 ± 0.007	0.6287
C18:3 *trans* isomer	0.130 ± 0.025 ^a^	0.261 ± 0.030 ^b^	0.0279
C18:3 all *cis*-9, 12, 15 (α-linolenic), n-3	2.74 ± 0.21 ^a^	3.63 ± 0.09 ^b^	0.0169
C20:0 (arachidic)	0.495 ± 0.012 ^B^	0.312 ± 0.010 ^A^	0.0003
C20:1 *cis*-11 (gondoic)	1.127 ± 0.023 ^B^	0.653 ± 0.026 ^A^	0.0002
C20:2 all *cis*-11, 14 (eicosadienoic), n-6	8.92 ± 0.46 ^B^	2.62 ± 0.07 ^A^	0.0002
C20:3 all *cis*-8, 11, 14 (dihomo-γ-linolenic), n-6	0.977 ± 0.198 ^B^	0.609 ± 0.006 ^A^	0.0095
C20:3 all *cis*-11, 14, 17 (eicosatrienoic), n-3	0.584 ± 0.114 ^B^	0.053 ± 0.006 ^A^	0.0097
C20:5 all *cis*-5, 8, 11, 14, 17 (eicosapentaenoic), n-3	3.323 ± 0.141 ^B^	0.169 ± 0.009 ^A^	0.0000
C21:0 (heneicosylic)	-	0.093 ± 0.006	
C22:0 (behenic)	0.193 ± 0.065	0.175 ± 0.009	0.7984
C22:1 *cis*-13 (erucic)	13.77 ± 0.45 ^B^	3.09 ± 0.21 ^A^	0.0000
C22:2 all *cis*-13, 16 (docosadienoic), n-6	0.068 ± 0.013	-	
C23:0 (tricosylic)	0.122 ± 0.048	0.077 ± 0.004	0.4129
C24:0 (lignoceric)	2.463 ± 0.147 ^B^	0.665 ± 0.043 ^A^	0.0003
**Fatty acid groups and ratios**			
Saturated fatty acids (SFA)	25.9 ± 0.4 ^B^	11.9 ± 0.8 ^A^	0.0001
Monounsaturated fatty acids (MUFA)	27.0 ± 0.3 ^A^	35.3 ± 1.4 ^B^	0.0044
Polyunsaturated fatty acids (PUFA)	33.6 ± 0.5 ^a^	35.7 ± 0.6 ^b^	0.0481
n-3	6.65 ± 0.20 ^B^	3.85 ± 0.07 ^A^	0.0002
n-6	26.5 ± 0.3 ^A^	31.4 ± 0.5 ^B^	0.0009
n-6/n-3	3.98 ± 0.09 ^A^	8.15 ± 0.03 ^B^	0.0000

^1^ Data are expressed as mean ± standard error of the mean (SEM). Statistically significant effect: values of one fatty acid without common superscript are statistically significantly different (^a,b^—at a significance level of *p* <0.05; ^A,B^—at a significance level of *p* < 0.01). Value “-“ means concentration <0.050 g/100 g fatty acid methyl esters (FAME). *n* = 3.

**Table 5:** Elements detected in lyophilised mucus, foot tissues and shells of *Helix aspersa* Müller ^1^.

Elements	Mucus	Foot Tissues	Shells	*p*
**Macroelements (g/kg Lyophilisate)**				
Na	70.26 ± 0.19 ^C^	6.53 ± 0.07 ^B^	1.08 ± 0.01 ^A^	0.0000
Ca	35.50 ± 0.12 ^B^	15.70 ± 0.79 ^A^	329.67 ± 0.88 ^C^	0.0000
K	9.67 ± 0.39 ^B^	9.98 ± 0.08 ^B^	1.77 ± 0.02 ^A^	0.0000
Mg	5.60 ± 0.04 ^C^	1.52 ± 0.02 ^B^	0.64 ± 0.01 ^A^	0.0000
P	1.87 ± 0.01 ^A^	9.50 ± 0.09 ^C^	2.56 ± 0.00 ^B^	0.0000
S	ND	5.01 ± 0.02 ^B^	1.11 ± 0.02 ^A^	0.0000
Cl	ND	1.56 ± 0.05 ^B^	0.41 ± 0.01 ^A^	0.0000
**Microelements (mg/kg lyophilisate)**				
Cu	239.00 ± 6.11 ^C^	29.67 ± 0.67 ^B^	10.33 ± 0.18 ^A^	0.0000
Zn	52.80 ± 2.21 ^B^	65.80 ± 0.76 ^C^	21.53 ± 0.54 ^A^	0.0000
B	32.43 ± 1.04 ^C^	7.95 ± 0.13 ^B^	1.42 ± 0.01 ^A^	0.0000
Fe	28.17 ± 0.93 ^A^	101.00 ± 0.58 ^B^	760.00 ± 10.69 ^C^	0.0000
Mo	6.81 ± 0.04 ^C^	2.62 ± 0.01 ^B^	0.03 ± 0.00 ^A^	0.0000
Mn	5.05 ± 0.37 ^A^	12.23 ± 0.19 ^B^	24.10 ± 0.21 ^C^	0.0000
Cr	3.45 ± 0.09 ^C^	2.45 ± 0.06 ^B^	0.11 ± 0.01 ^A^	0.0000
Ni	1.64 ± 0.06 ^A^	9.49 ± 0.14 ^C^	2.33 ± 0.08 ^B^	0.0000
Se	0.27 ± 0.06	0.36 ± 0.03	-	0.2114
Co	0.10 ± 0.00 ^B^	0.07 ± 0.00 ^A^	0.14 ± 0.00 ^C^	0.0000
Si	ND	820 ± 20.82 ^B^	123.33 ± 3.18 ^A^	0.0000

^1^ Data are expressed as mean ± standard error of the mean (SEM). Statistically significant effect: values of one element without common superscript (^A,B,C^) are statistically significantly different (*p* < 0.01). Value “-” means concentration <0.05 mg/kg lyophilisate for Se; ND—element which was not determined in mucus. *n* = 3.
